# Looking forwards and backwards: The real-time processing of Strong and Weak Crossover

**DOI:** 10.5334/gjgl.280

**Published:** 2017-07-31

**Authors:** Dave Kush, Jeffrey Lidz, Colin Phillips

**Affiliations:** 1 Norwegian University of Science and Technology (NTNU), NTNU, Institutt for språk og litteratur, N-7491 Trondheim, NO; 2 Haskins Laboratories, 300 George St., New Haven, CT 06511, US; 3 Department of Linguistics, University of Maryland, 1401 Marie Mount Hall, College Park, MD 20742, US

**Keywords:** crossover, antecedent retrieval, c-command, sentence processing, syntactic prediction

## Abstract

We investigated the processing of pronouns in Strong and Weak Crossover constructions as a means of probing the extent to which the incremental parser can use syntactic information to guide antecedent retrieval. In Experiment 1 we show that the parser accesses a displaced wh-phrase as an antecedent for a pronoun when no grammatical constraints prohibit binding, but the parser ignores the same wh-phrase when it stands in a Strong Crossover relation to the pronoun. These results are consistent with two possibilities. First, the parser could apply Principle C at antecedent retrieval to exclude the wh-phrase on the basis of the c-command relation between its gap and the pronoun. Alternatively, retrieval might ignore any phrases that do not occupy an Argument position. Experiment 2 distinguished between these two possibilities by testing antecedent retrieval under Weak Crossover. In Weak Crossover binding of the pronoun is ruled out by the argument condition, but not Principle C. The results of Experiment 2 indicate that antecedent retrieval accesses matching wh-phrases in Weak Crossover configurations. On the basis of these findings we conclude that the parser can make rapid use of Principle C and c-command information to constrain retrieval. We discuss how our results support a view of antecedent retrieval that integrates inferences made over unseen syntactic structure into constraints on backward-looking processes like memory retrieval.

## 1 Introduction

During incremental sentence processing the parser routinely engages backward-looking processes in order to establish a dependency between an item in the current input and an item that was previously seen. Establishing a dependency between either *the parents* or *which boys* for the pronoun *them* in (1) is one such process.

(1) **The parents** wondered **which boys** thought that the girl was laughing at **them**.

It is commonly assumed that the preliminary stage of this process requires *retrieving* a representation of the antecedent from a store in memory (Sanford & Garrod 1981; Gordon & Hendrick 1998; Sanford & Garrod 1998; Kennison 2003; Foraker & McElree 2007). Many prominent models of retrieval posit that items in memory are identified according to their feature content (Nairne 1990; McElree 2000; Lewis, Vasishth & Van Dyke 2006). In the context of antecedent retrieval for anaphora, this is most commonly translated into the assumption that candidate antecedents are identified on the basis of morphological feature-match with a pronoun (e.g. Arnold et al. 2000; though see Cunnings, Patterson & Felser 2014). In its simplest form, morphologically driven antecedent retrieval should allow access to any NP that both matches and precedes a pronoun, which guarantees access to an antecedent whenever one is present. However, such a procedure would have the undesirable property of allowing the parser to consider antecedent-pronoun relations that are not sanctioned by the grammar. Syntactic research has demonstrated many situations where a preceding and feature-matching NP is prohibited from serving as an antecedent for a pronoun. Grammatical constraints rule out such NPs (which we term *distractor NPs*) on the basis of properties of the larger representation in which they are contained. Most syntactic constraints reference the relative geometric position of the preceding distractor and the pronoun. For example, the Bound Anaphor Condition (Reinhart 1983) bars the quantified NP (QP) *no boy* from binding the matching pronoun *him* in (2) because the QP does not c-command the pronoun from its position inside the relative clause. That is, the pronoun is not contained within the QP's sister phrase in the syntactic tree.

(2) *The girl [that **no boy_i_** talked to] tried to speak to **him_i_**.

Although properties of the syntactic representation play an integral role in accounts of untimed binding interpretations, less is known about which of these properties are used to guide immediate retrieval of potential antecedents during incremental parsing. There are differing views on the degree of alignment between grammatically acceptable representations and the representations constructed during online comprehension (Ferreira & Patson 2007; Lewis & Phillips 2015). Also, even in a world where online processes are tightly coupled to grammatical constraints, it is possible that grammatical constraints apply as a filter on a broader initial set of representations, paralleling the assumption of many grammatical models (Chomsky 1981; Grimshaw 1997; Legendre, Smolensky & Wilson 1998).

Recent studies have made progress on identifying certain properties that are available to retrieval by studying the real-time application of grammatical constraints. We highlight two lines of investigation that illustrate the scope of current knowledge.

One line of research suggests that antecedent retrieval can exploit local c-command relations between a distractor and a subsequent pronoun. A number of studies (Badecker & Straub 2002; Kennison 2003; Chow, Lewis & Phillips 2014) have tested comprehenders' immediate sensitivity to Principle B (Chomsky 1981; Reinhart 1983; Grodzinsky & Reinhart 1993) using a gender-mismatch paradigm such as the one in (3). The experiments manipulated the gender match between a pronoun (*him*) and two preceding NPs: (i) a non-local NP (*Arthur/Anne*) that could serve as a structurally appropriate NP for the pronoun and (ii) a potential distractor NP (*Jane/Bill*). The distractor could not serve as an antecedent, according to Principle B, because it was a c-commanding clause-mate of the pronoun.

(3)

- Arthur thought that Jane should give **him** another chance.
- Arthur thought that Bill should give **him** another chance.
- Anne thought that Jane should give **him** another chance.
- Anne thought that Bill should give **him** another chance.

Across all previous studies, participants have consistently exhibited greater difficulty processing the pronoun in sentences in which there was no grammatically acceptable NP that matched the pronoun in gender (3c, d) compared to sentences in which there was a matching grammatically acceptable NP (as in (3a, b)). The fact that the pronoun is more difficult to process in (3d) than in (3a, b) is consistent with the assumption that retrieval favors NPs that are acceptable antecedents according to Principle B over those that are not. A parser that did not make the relevant distinction would be expected to initially process the pronoun in (3d) as easily as the pronoun in (3a, b) on the assumption that morphological feature-match alone would lead the parser to temporarily consider the local NP *Bill* as a potential antecedent.

Although researchers generally agree that grammatical antecedents enjoy a retrieval advantage, there is less consensus on whether retrieval nevertheless considers unacceptable antecedents to some degree. Badecker & Straub (2002) observed that processing of the pronoun was more difficult in (3b) than in (3a), and took this to indicate that the unacceptable NP *Bill* interfered with retrieval of *Arthur* as the antecedent of the pronoun. The authors interpreted this *inhibitory interference* as evidence that the parser occasionally retrieved *Bill* due to morphological match. However, the researchers found no evidence that *Bill* facilitated processing of the pronoun in (3d), relative to (3c). Such *facilitatory interference* would have been expected if the parser were prone to misretrieve *Bill* as an antecedent. More recent work (Chow, Lewis & Phillips 2014), has failed to find evidence of either inhibitory or facilitatory interference, suggesting that antecedent retrieval does not consider antecedents ruled out by Principle B.

A complementary line of research has asked whether the parser can use non-local c-command relations to guide retrieval (Kush, Lidz & Phillips 2015; Cunnings, Patterson & Felser 2015). In a series of experiments Kush, Lidz & Phillips (2015) tested if the parser would access a quantificational NP (QP) as an antecedent for a pronoun that it did not c-command, in violation of the c-command condition on quantifier-variable binding (Reinhart 1983). Those experiments manipulated the gender-match between a distractor QP embedded within a relative clause (*no girl/boy scout* in (4a)) and a pronoun within a higher clause (*her*). Reading times at the pronoun in (4a) were compared to the pair in (4b), where the embedded phrase was referential (*the girl/boy scout*). Because coreference does not require c-command, the authors reasoned that retrieval should have access to the referential NP in (4b) when it matched the pronoun.

(4)

- The troop leaders that no **girl**/boy scout had respect for scolded **her** after …
- The troop leaders that the **girl**/boy scout had no respect for scolded **her** after …

Kush and colleagues found that gender match between the antecedent NP and the pronoun clearly facilitated processing of the pronoun when the antecedent was referential, but not when the antecedent was quantificational. The authors therefore concluded that comprehenders easily retrieved the referential NP as an antecedent for the pronoun, but did not retrieve the QP on account of its position.

Taken together, prior research indicates that the parser can use the geometric relation between a pronoun and a preceding NP to constrain antecedent retrieval.

In this paper we seek to further explore the parser's ability to marshal syntactic information to prevent the retrieval of distractors. We investigate the processing of pronouns in Crossover configurations (Postal 1971; Wasow 1972), which we illustrate using the *Strong Crossover* construction in (5). In cases of Crossover the displaced wh-phrase *which girl* (henceforth, the *wh*-*filler*) is grammatically prohibited from binding the pronoun *she* despite the fact that the filler both matches the pronoun in morphological features and c-commands it from a position outside its local clause.

(5) Bob asked which girl it seemed that **she** thought Bill made fun of ___.

Confgurations of this sort have been dubbed ‘crossover’ constructions because the path that links the wh-filler to its gap position (indicated by the underscore) ‘crosses over’ the pronoun's linear position. The unacceptability of binding in (5) contrasts sharply with the general acceptability of wh-filler antecedents in configurations like (6) where crossover does not occur.

(6) Bob asked **which girl**___ had said that **she** thought Bill made fun of Clint.

Crossover constructions provide a valuable test case of the limits of syntax-guided retrieval, because the relative surface geometric configuration between the distractor and the pronoun is insufficient to rule out binding, unlike in previous constructions tested. If retrieval's ability to draw on syntactic information is limited to using the geometry of the preceding representation, then we would expect the parser to (temporarily) consider *which girl* as a binder of the pronoun in (5). If, however, antecedent retrieval avoids interference from the unlicensed wh-filler in Crossover configurations, we would have evidence that retrieval processes have a wider range of syntactic information at their disposal. As such, the first goal of our study was to determine whether the parser is subject to interference from the distractor *which girl* in Strong Crossover constructions like (5). To preview our results, we find evidence that retrieval is sensitive to the distinction between unacceptable fillers in Strong Crossover configurations and acceptable fillers that can bind a pronoun in non-Crossover configurations.

In light of the sensitivity of retrieval to Strong Crossover, we ask a second question: what property of the syntactic representation does retrieval exploit to exclude consideration of the unacceptable filler? Grammatical accounts of crossover link the unacceptability of Strong Crossover to two distinct constraints, each of which targets a different aspect of the syntactic representation.

The unacceptability of binding in Strong Crossover is commonly blamed – at least in part – on a violation of Binding Principle C (Chomsky 1982). Like other constraints on anaphora, Principle C makes reference to the geometry of the syntactic representation to rule out binding. However, unlike more common constraints on anaphora, which regulate binding based on superficial relations between an anaphor and a preceding item, Principle C rules out binding in Strong Crossover on the basis of the relation between the pronoun and the gap position of the filler, which follows the pronoun in the linear string. Principle C violations in Strong Crossover are easy to diagnose offline once the full structure of a sentence has been parsed, but identification is potentially more complicated for an incremental parser that does not have access to the entire sentence at the time it first encounters the pronoun. During left-to-right processing of Crossover constructions the parser encounters the pronoun before it has bottom-up confirmation of the gap position (as illustrated in (7)).

(7) Bob asked which girl it seemed that **she …**

If the parser used knowledge of Principle C to block access to the feature-matching filler in (7) the action would have to be made based on the prediction that the filler's gap must fall within the c-command domain of the pronoun (as illustrated in (8)).

(8) Bob asked which girl_i_ it seemed that **she** [… ____i_]


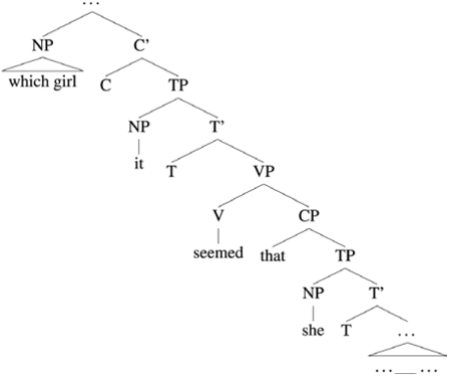


Prior work (Kazanina et al. 2007) has demonstrated that knowledge of Principle C can be used to guide prospective search for antecedents during cataphoric processing, but the configurations tested in those experiments did not require antecedent retrieval. Thus, it is unknown at present whether inferences over yet-to-be-seen structural relations can be used to constrain antecedent retrieval. As we discuss further in the General Discussion, forward-looking sensitivity to Principle C poses an interesting challenge for retrieval models that assume that retrieval is exclusively driven by properties of the prior context (e.g. Lewis, Vasishth & Van Dyke 2006).

It is also possible that the parser could rule out binding in (8) without direct reference to the configuration of the pronoun and the gap. In addition to Principle C, researchers assume that Strong Crossover is also governed by a constraint that does not make direct reference to c-command (Chomsky 1976; Koopman & Sportiche 1982; Reinhart 1983; Sportiche 1985; Ruys 2000; Shan & Barker 2006). Motivation for this constraint comes from *Weak Crossover* constructions like (9). Binding in (9) is not prohibited by Principle C, because the pronoun *her* does not c-command the gap, nevertheless the relation is unacceptable.^1^

(9) Bob asked which girl it seemed that **her** friend thought Bill made fun of ___.

Researchers disagree on the precise formalization of the constraint responsible for these judgments, but most accounts link the unacceptability of filler-pronoun binding in (8) and (9) to the fact that the filler occupies a non-argument position (the specifier of CP). For example, many syntactic accounts require that an NP must occupy an A(rgument)-position in order to bind a pronoun (Reinhart 1983; Ruys 2000). Because fillers in Strong and Weak Crossover configurations do not meet this criterion, they are not eligible binders.

The requirement that binding must occur from an A-position would be straightforwardly captured if antecedent retrieval were “blind” to any phrase in a non-argument position. Filler-blind retrieval would easily avoid interference from matching wh-phrases in both Strong and Weak Crossover configurations. However, such a parser would also need to ensure that fillers in acceptable configurations such as (6) were somehow made “visible” to retrieval before the pronoun was encountered. Intuitively, this could be achieved by updating the representation of a filler so that it was treated like an ordinary argument phrase once its gap had been encountered during left-to-right parsing. This would allow the filler in (6) to essentially bind the pronoun from the gap position (Shan & Barker 2006).^2^

Such an implementation would constitute evidence that in addition to the geometry of the preceding representation, retrieval must also have information about the type of syntactic position occupied by a phrase. We note that this solution would appear intuitively preferable to an explanation in terms of Principle C violations for two reasons. First, it would have wider empirical coverage, encompassing both cases of Strong and Weak Crossover. Second, it would link the accessibility of the filler to a superficial property of the preceding representation, a conclusion that aligns with the widely shared assumption that retrieval uses aspects of an item's local (encoding) context that can be coded as content features for cue-based memory access (see Lewis, Vasishth & Van Dyke 2006; Kush, Lidz & Phillips 2015).

Because (9) and (5) are identical in all relevant respects except for the pronoun-gap c-command relation, the A-binding constraint that blocks binding in (9) also applies to Strong Crossover configurations. Therefore, it would not be possible to determine, based on sensitivity to Strong Crossover alone, which aspect of the syntactic representation bore responsibility for excluding the filler immediately at retrieval.

In the experiments described below we sought to tease apart these two options by comparing interference in Strong and Weak Crossover constructions. We reasoned that the processing of pronouns in Weak Crossover configurations should provide a measure of sensitivity to the constraint on A-binding. If antecedent retrieval is equally good at ignoring matching wh-fillers in Weak and Strong Crossover configurations, then we would have evidence that retrieval is sensitive to the type of position that its targets occupy. We would have no evidence for Principle C sensitivity. If, on the other hand, we find that antecedent retrieval is better at resisting the influence of matching wh-fillers in Strong Crossover than in Weak Crossover, we can attribute the residual difference to Principle C.

## 2 Experiment 1: Strong Crossover

Experiment 1 measured the impact of a feature-matching wh*-*filler on the processing of a subsequent pronoun using a gender-mismatch paradigm. We compared the effect of the gender match between the filler and the pronoun in sentences in which the filler was an acceptable antecedent for the pronoun to sentences in which the filler and pronoun were in a Strong Crossover configuration.

### 2.1 Materials

Experimental materials consisted of 36 sets of 6 conditions each; an example set of test items is provided in Table 1.

All test items were a single sentence composed of three right-branching clauses. The critical pronoun was the subject of the most deeply embedded clause. The wh-filler occupied the left periphery of the second clause, creating an indirect question. We chose to use indirect questions instead of direct questions to minimize pragmatic load and to provide a small amount of context for the critical clauses. This design choice also made it possible to ask participants yes/no comprehension questions. The gender of the main clause subject never matched the gender of the pronoun, so coreference between the two was not possible.

Four of the six conditions followed a 2 × 2 factorial design crossing the factors GenderMatch and Crossover. The GenderMatch factor manipulated the gender of the wh-filler so that it matched or mismatched the pronoun in gender. We held the gender of the pronoun constant across test items and manipulated GenderMatch by varying the head NP of the wh-filler. Head nouns were either definitionally or stereotypically gendered. Both noun types have been shown to produce reliable gender-mismatch effects (Sturt 2003; Kreiner, Sturt & Garrod 2008).

The factor Crossover manipulated whether the filler was linked to a gap that preceded or followed the pronoun. In the NoCrossover conditions, the gap was in the subject position of the second clause, where it preceded and c-commanded the pronoun. Hence, the filler was a structurally appropriate antecedent. In the Crossover conditions the gap was the object of a post-verbal preposition in the third clause. In this position the gap followed and was c-commanded by the critical pronoun. In this configuration, the filler was not a grammatically acceptable antecedent for the pronoun.

The second clause predicate also varied with the Crossover factor. In NoCrossover sentences the second clause predicate was a manner of speech verb or a propositional attitude verb. In Crossover sentences, the predicate was a raising predicate (*it seemed/it appeared*). Raising predicates were used because they permit expletive subjects that do not add to the referential load or complexity of the intervening clause (Gibson 1998). This allowed us to hold referential complexity constant across Crossover and NoCrossover conditions.

Two control conditions were added to the basic 2 × 2 design. These control conditions were identical to the Crossover-Match and NoCrossover-Match conditions with one change. In Control conditions, the subject pronoun in the third clause was replaced by a proper name (*Dana* in Table 1). These conditions provide a baseline of processing complexity that factors out the contribution of pronoun processing. For example, they allow us to measure any processing costs associated with the different filler-gap dependencies computed in Crossover and NoCrossover sentences. The fillers also served an additional practical purpose: they disrupted participants' ability to predict a downstream pronoun upon reading an intermediate wh-phrase.

Based on prior research, we expected a gender-mismatch effect in the NoCrossover conditions (Garnham et al. 1995; Badecker & Straub 2002). We expected participants to have no difficulty processing the critical pronoun in the NoCrossover-Match condition because the matching filler could serve as an acceptable antecedent. We expected participants to experience greater processing difficulty at the pronoun in the NoCrossover-Mismatch condition because the pronoun would lack an explicit matching antecedent (Osterhout & Mobley 1995). We expected the gender-mismatch effect to manifest in judgment studies as a decrease in the average acceptability of NoCrossover-Mismatch condition compared to the NoCrossover-Match condition. In the self-paced reading studies we expect longer RTs in NoCrossover-Mismatch condition either immediately at the pronoun or in the next region, as it is common for such mismatch effects to be found in one or two “spillover” regions directly following the critical word or region of interest (Just & Carpenter 1978).

We assessed the sensitivity to the Strong Crossover constraint by measuring whether gender-match had a similar effect on the processing of the pronoun in Crossover sentences. If either Principle C or the A-binding constraint acts as an immediate categorical restriction on antecedent retrieval, we would predict that there should be no gender-mismatch effect in Crossover sentences. Average acceptability ratings should not differ in our judgment studies and there should be no reliable difference in RTs between the Crossover-Match and Crossover-Mismatch conditions at the pronoun or spillover region in the online reading record. If, however, neither constraint applies at the point of antecedent retrieval, we should observe a gender-mismatch effect in the Crossover sentences that is comparable in size (and location) to the gender-mismatch effect in the NoCrossover sentences.

### 2.2 Experiment 1a

We first conducted an offline acceptability judgment task to verify that speakers rejected Strong Crossover violations in our test sentences.

#### 2.2.1 Participants

Twenty-two participants (mean age = 36.2, range 21–60, 6 male) were recruited through the Amazon Mechanical Turk (AMT) marketplace and paid $4.00 for their participation (see Gibson, Piantadosi & Federenko 2011 and Sprouse 2011 for discussion of collecting judgments on AMT). Participants' IP addresses were restricted to US-internal locations. Three participants were excluded from analysis because their average ratings did not differ across conditions, suggesting that they did not perform the task as requested.

#### 2.2.2 Procedure and materials

Presentation used the IBEX Farm web-based experimental presentation platform, developed and managed by Alex Drummond (www.spellout.net/ibexfarm/docs). Sentences were presented above a 7-point acceptability scale. The left and right end-points of the scale were labeled ‘bad’ and ‘good’, respectively. Participants could record a response either by using their cursor to click a value, or by pressing a number on their keyboard. Participants were given three practice sentences prior to beginning the exercise. Test items were interspersed among 60 acceptable fillers of comparable length and complexity.

#### 2.2.3 Analysis

Acceptability ratings from individual participants were z-scored prior to analysis. We ft linear mixed-effects models to the ratings data using the lmerTest package (Kuznetsova, Brockhof & Christensen 2015) in R (R Development Core Team). Models included a simple difference coded fixed effect of Crossover, a Helmert-coded fixed effect of ConditionType, and their interaction. The helmert-coded effect of ConditionType had three levels: *Control v. Test, GenderMatch*, and *GenderMismatch*. Coding ConditionType in this fashion allowed us to isolate two different contrasts of interest. The first contrast compared the mean value of the dependent variable (rating or RT) in the control conditions to mean value of the pronoun conditions (we label this effect Pro vs. Control in subsequent discussion). The second contrast measured the effect of GenderMatch within the pronoun conditions by comparing the mean values of sentences with a matching pronoun to those with a mismatching pronoun.

We used ‘parsimonious’ random effects structures for all models, as recommended by Bates et al. (2015). Random effects structures were determined by first fitting a model with a maximal random effects structure (Barr et al. 2013) and then iteratively simplifying the model by removing random effects with zero variance identified using the rePsych-Ling package (Bates et al. 2015). Reported p-values were extracted from the fitted model objects using the Satterthwaite approximation implemented by the *lmerTest* package (Kuznetsova et al. 2015). Pairwise comparisons were conducted using the difflsmeans() function in lmerTest.

#### 2.2.4 Results and discussion

Average raw and z-scored acceptability judgment scores are given in Table 2. A summary statistical analysis is in Table 3.

Crossover sentences were rated higher on average than NoCrossover sentences (*p* < .001), control sentences received higher average ratings than test sentences (*p* < .001), and Match sentences were rated higher than NoMatch sentences (*p* < .001). The higher ratings in the control conditions indicate that (i) the filler-gap dependencies themselves were not a source of unacceptability in either Crossover or NoCrossover sentences were acceptable and (ii) remaining differences in acceptability should be linked to the presence of the pronoun. The significant Crossover × Pro v. Control (*p* < .001) interaction reflected the fact that Crossover had a more pronounced effect on test conditions than on control conditions.

The Crossover × GenderMatch (*p* < .001) interaction indicates that the effect of gender match between the pronoun and the filler was different between Crossover and NoCrossover sentences. Gender match between the filler and the pronoun resulted in higher acceptability scores in the NoCrossover conditions (*t* = 10.38, *p* < .001), but had no discernible effect on the rating of the Crossover conditions (*t* < 1). This statistical analysis corroborates what is clear from the ratings in Table 2: among the test conditions NoCrossover-Match condition received the highest ratings, consistent with the interpretation that participants easily established a dependency between the filler and the pronoun. When the sentences contained a mismatching filler in the same position, acceptability scores were lower, confirming that an antecedent-less pronoun reduces the acceptability of test sentences (Gordon & Hendrick 1997). Crossover sentences received relatively low acceptability ratings. This pattern of acceptability scores confirms that participants display offline sensitivity to Strong Crossover.

### 2.3 Experiment 1b: self-paced reading

Experiment 1a confirmed that native speakers reject binding between wh-fillers and pronouns in our Strong Crossover materials, indicating that our constructions were suitable for testing the real-time application of the constraint. In Experiment 1b we tested the effect of Strong Crossover in incremental comprehension by measuring whether illicit antecedents are initially considered upon encountering a pronoun.

#### 2.3.1 Participants

Fifty participants from the University of Maryland community participated in the experiment in exchange for course credit.

#### 2.3.2 Procedure

Participants were run on a desktop PC using the Linger software package (Doug Rohde, MIT) in a self-paced word-by-word moving window paradigm (Just, Carpenter & Woolley 1982). Each trial began with a sentence masked by dashes appearing on the screen. Letters and punctuation marks were masked, but spaces were left unmasked so that word-boundaries were visible. As the participant pressed the spacebar, a new word appeared on the screen and the previous word was re-masked. Test sentences were pseudo-randomly interspersed among 90 filler sentences of comparable length and complexity. A comprehension question followed each sentence. Participants were instructed to read sentences at a natural pace and to respond to the comprehension questions as accurately as possible. Participants responded to questions using the f-key for ‘yes’ and the j-key for ‘no.’ Participants were notified if they answered incorrectly. Each participant was randomly assigned to one of the six lists, and the order of the stimuli within each presentation list was pseudo-randomized so that no two test items were ever presented in direct succession.

#### 2.3.3 Analysis

Two participants' data were excluded from analysis for having abnormal RT variance.^3^ Prior to analysis we decided upon 70% accuracy on comprehension questions as a threshold for inclusion in further analysis. All participants exceeded this threshold; thus none were excluded. Reaction times (RTs) above 2.5 standard deviations of the mean by region and condition were excluded from analysis (Ratcliff 1993), resulting in an exclusion of roughly 2.7% of all observations. Statistical analyses of accuracy data and log-transformed RTs used mixed-effect models with the structure described in Experiment 1a.

We present analysis of RTs in a number of regions of interest: the pre-pronoun *complementizer*, the critical pronoun, as well as the *adverb,* and *verb*. We note, however, that we base our conclusions about the presence or absence of a gender-mismatch effect on the RTs at the pronoun and post-pronoun *adverb* region (the spillover region). The pre-pronoun region was presented and analyzed simply to establish that there were not pre-pronoun differences between the conditions that could have confounded measurement of the effect of gender-mismatch.

#### 2.3.4 Results

##### Comprehension question accuracy

The mean comprehension question accuracy for experimental items across participants was 93.3%. Accuracy did not differ significantly across conditions (logistic mixed effects model, all *z* values < 1).

##### Self-paced reading results

**Figure 1:**
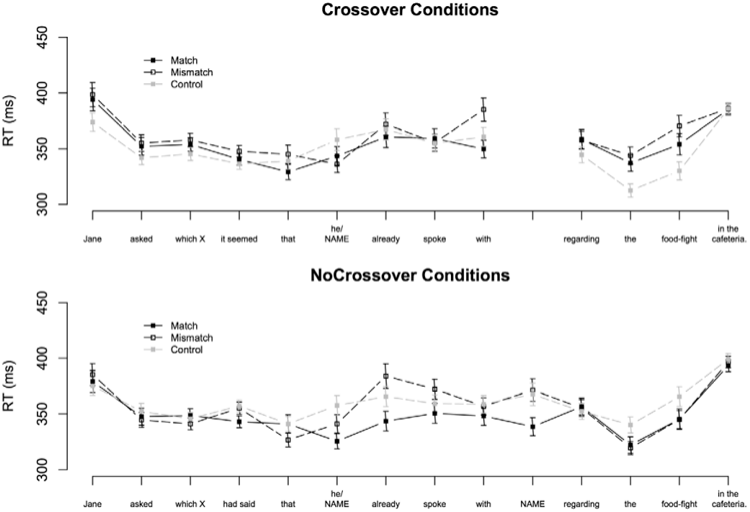
Average self-paced reading times Experiment 1b. Error bars indicate standard error of the cell means.

Average word-by-word RTs by region and condition are plotted in Figure 1.^4^ A summary of the statistical analyses of test conditions is shown in Table 4.

There were no significant effects at the pre-critical complementizer. At the critical pronoun, the Control conditions were read more slowly than the Pronoun conditions (*t* = 2.72, *p* < .01). This difference presumably reflects the fact that participants took longer to read proper names than pronouns.

At the post-pronoun adverb the model revealed a significant main effect of GenderMatch (*t* = –3.16, *p* < .01), which was qualified by a marginally significant Crossover × GenderMatch interaction (*t* = –1.87, *p* < .10). Planned comparisons showed a clear gender-mismatch effect in the NoCrossover conditions: gender match between the filler and the pronoun facilitated processing (*t* = 3.55, *p* < .001; difference = 40 ms). The small numerical trend towards a gender-mismatch effect in the Crossover conditions was not statistically reliable (|*t*| < 1). A marginally significant Crossover × GenderMatch interaction was also observed at the following verb region (*t* = 1.77, *p* < .10). Planned comparisons again revealed a significant gender-mismatch effect in the NoCrossover conditions (*t* = 2.22, *p* < .05; difference = 22 ms). Gender match did not facilitate processing in the Crossover conditions (*t* < 1; difference = –4 ms).

#### 2.3.5 Discussion

Experiment 1 tested whether retrieval accessed a feature-matching but grammatically unacceptable wh-filler as a potential antecedent for a critical pronoun in Strong Crossover constructions. We determined whether the wh-filler had been retrieved by measuring the effect that gender-match with the wh-filler had on the processing of the pronoun. We compared the effect of the filler when its gap came before the pronoun to its effect when the pronoun preceded and c-commanded the gap. When the gap preceded the pronoun the filler was a grammatically acceptable antecedent, but when the gap followed the pronoun the filler was not an acceptable antecedent because binding was ruled out by both Principle C and the A-binding constraint.

Offline ratings aligned with the grammatical generalization that speakers reject binding under Strong Crossover. Participants gave high ratings to NoCrossover sentences when the filler matched the pronoun in gender, and gave low ratings to NoCrossover sentences when the filler mismatched the pronoun. Crossover sentences received low ratings regardless of whether the filler matched the pronoun.

The self-paced reading results immediately following the pronoun reflected a pattern similar to the offline judgments. Reading times two regions after the pronoun were faster when the filler matched the pronoun in gender and the two were not in a Crossover configuration. When the filler and the pronoun were in a Strong Crossover configuration, gender match did not have a reliable effect on processing in the same regions. We acknowledge that the full Crossover × GenderMatch interaction was only marginally significant in the full analysis, but we do not take this as evidence against Crossover sensitivity. Planned comparisons revealed differences between the size of the gender mismatch effect in the Crossover and NoCrossover sentences. The negligible difference between reading times in the matching and mismatching Crossover conditions also strongly suggests that participants did not consider matching wh-fillers in Strong Crossover configurations. In light of the close alignment between the grammatical generalization, the offline judgments in Experiment 1a, and the reading times in Experiment 1b, it is unlikely that pronoun processing ignores Crossover constraints. The results are consistent with either of the following possibilities: either Crossover constraints apply categorically or as highly-weighted constraints. We return to this point our General Discussion.

Overall, the results of Experiment 1 indicate that antecedent retrieval displays immediate alignment with offline judgments of binding relations in Strong Crossover configurations. However, our results do not determine which grammatical constraint is responsible for this alignment. In Experiment 2 we investigate the processing of Weak Crossover constructions to disentangle the contributions of Principle C and the A-binding constraint.

## 3 Experiment 2: Weak Crossover

Experiment 1 demonstrated that the presence of a matching wh-filler had little, if any, impact on the immediate processing of the critical pronoun if the filler and the pronoun were in a Strong Crossover configuration. Experiment 2 sought to determine whether a matching filler would be similarly ignored at a critical pronoun when the pronoun and filler were in a Weak Crossover configuration. If fillers are ignored in both configurations, it would suggest that the parser uses the non-argument status of the wh-filler to exclude binding under Crossover. If pronoun resolution appears to consider matching fillers in Weak Crossover configurations, we would have evidence that retrieval does not make immediate use of information about position type. Under this scenario the results of Experiment 1 would provide evidence that Principle C is responsible for the sensitivity shown in Experiment 1, and hence that pre-computation of relevant structure must be involved.

### 3.1 Materials

The materials from Experiment 1 were changed minimally to create Weak Crossover configurations. The critical subject pronouns from Experiment 1 were replaced with NPs with pronominal possessors (e.g., *he* in Experiment 1 was changed to *his supervisor* in Experiment 2). In this configuration the critical pronoun does not c-command the gap site, because it is embedded within the larger subject phrase.

The proper names used in the embedded subject position in the control conditions of Experiment 1 were replaced with definite NPs whose head noun matched the possessed NP in the test conditions. This was done to ensure that the embedded subject region was as closely matched as possible across the test and control conditions. The post-pronoun critical region was also lengthened by adding an additional auxiliary between the critical subject NP and the verb. An example item set is shown in Table 5.

### 3.2 Experiment 2a: Acceptability judgment

We conducted an acceptability judgment study to verify that the wh-fillers in Weak Crossover configurations would be judged unacceptable.

#### 3.2.1 Participants

Twenty-one participants (mean age = 33.6, 6 male) were recruited through the Amazon Mechanical Turk marketplace, and paid $4.00 for their participation. Participants were recruited using the same criteria as for Experiment 1a. Three participants were excluded from analysis because their ratings of control sentences were unusually low.

#### 3.2.2 Methods and analysis

Experimental presentation and analysis were identical to Experiment 1a. The 36 experimental items from Experiment 2 were interspersed among 30 acceptable and 26 unacceptable fillers.

#### 3.2.3 Results and discussion

Raw and z-scored acceptability judgment scores are given in Table 6. Summary statistical analysis is presented in Table 7.

As in Experiment 1a, NoCrossover sentences were rated higher on average than Crossover sentences, Control sentences received higher ratings than test sentences, and Match sentences were more acceptable than Mismatch sentences (all *p*s < .001).

The average acceptability ratings exhibit a pattern indicative of Weak Crossover sensitivity, similar to the Strong Crossover sensitivity observed in Experiment 1a. There was a robust GenderMatch × Crossover interaction (*t* = 3.74, *p* < .001), which indicates that gender match affected Crossover and NoCrossover sentences differently. Gender match had a reliable effect on the acceptability of the NoCrossover sentences (*t* = 6.31, *p* < .001). The mean acceptability of the Match-Crossover sentences was slightly higher than NoMatch-Crossover sentences, but this small numerical difference was not significant in planned comparison (*t* = 1.01). It is possible that with greater power that this numerical difference would achieve significance, which in turn would lend quantitative support to the general intuition that Weak Crossover is “weaker” or more easily violated than Strong Crossover (e.g. Lasnik & Stowell 1991). Overall, however, the principal finding of Experiment 2a was that participants find violations of Weak Crossover unacceptable, irrespective of how this small difference should be interpreted.

### 3.3 Experiment 2b: Self-paced reading

#### 3.3.1 Participants

Thirty participants from the University of Maryland community participated in the experiment in exchange for course credit.

#### 3.3.2 Procedure

**Figure 2:**
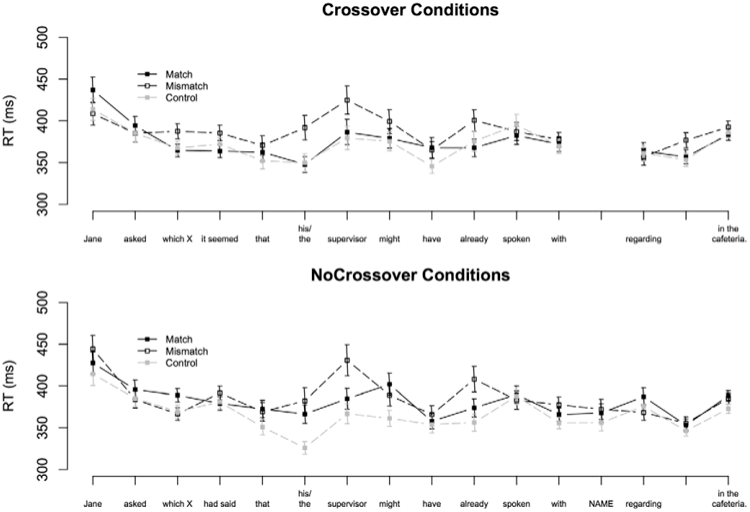
Average word-by-word self-paced reading times Experiment 2b. Error bars indicate standard error of the cell means.

The procedure was identical to Experiment 1b. The pre-pronoun region of interest in Experiment 2b was the same as in Experiment 1b. As in Experiment 1b, we present analysis from a number of words between the pronoun and the preposition immediately following the verb. In Experiment 2b these regions are: the post-pronoun noun (*supervisor* in Figure 2), and the *first auxiliary* (*might*).

#### 3.3.3 Analysis

The data of one participant were excluded due to history of cognitive impairment. One participant whose average accuracy on comprehension questions fell below a 70% cut-of threshold on all items (fillers and test items) was excluded from analysis. One item was excluded due to a typo. Statistical analysis was identical to Experiment 1b.

#### 3.3.4 Results

##### Comprehension question accuracy

Mean comprehension question accuracy on test items across participants was 83.0%. Accuracy did not differ significantly across conditions (|*z*| < 1 for all effects).

##### Self-paced reading results

Average word RTs by region and condition are plotted in Figure 2. A statistical summary of all results is given in Table 8.

There was a significant GenderMatch × Crossover interaction in the wh-filler region (*t* = –2.92, *p* < .01). Matching fillers were read significantly more slowly than mismatching fillers in the NoCrossover conditions (*t* = 2.43, *p* < .05). A marginally significant difference in the opposite direction was observed in the Crossover conditions (*t* = –1.70). We consider this effect spurious and attribute it to experimental noise because all conditions were lexically identical up to and including the wh-filler region. In the following region Crossover sentences were read more slowly than NoCrossover sentences (*t* = –2.19). This pattern appears to be driven by a sustained slowdown in the Crossover-Mismatch condition. This numerical difference carried over into the pre-pronoun complementizer region, but differences between conditions were not significant.

Our model revealed that the Control conditions were read more quickly in the pronoun region than the Pronoun conditions (*t* = –3.45, *p* < .01). Among the pronoun conditions, the Match conditions were read marginally more quickly on average than the Mismatch conditions (*t* = 1.66, *p* < .10). Somewhat surprisingly, the gender-mismatch effect was greater in the Crossover conditions than in the NoCrossover conditions, as indicated by a marginally significant GenderMatch × Crossover interaction (*t* = 1.81, *p* < .10). Planned comparisons revealed a significant gender-mismatch effect in the Crossover conditions (*t* = 2.46, *p* < .05), but not in the NoCrossover conditions (*t* < 1). It is important to note that this GenderMatch × Crossover interaction reflects a pattern that is the opposite of what we would have expected to observe if antecedent retrieval were sensitive to the A-binding constraint.

Reading times at the post-pronoun nominal were faster when the filler matched the pronoun than when it mismatched it (*t* = 2.75, *p* < .01). At the first auxiliary following the critical region there was a marginally significant GenderMatch × Crossover interaction (*t* = –1.88, *p* < .10). Once again this interaction reflected a trend towards a larger gender-mismatch effect in the Crossover conditions than in the NoCrossover conditions, but the gender-mismatch effect in the Crossover conditions did not prove to be reliable in planned comparisons (*t* = 1.25).

#### 3.3.5 Discussion

The significant gender-mismatch effects at the pronoun and on the following noun provide preliminary evidence that the argument/non-argument status of the filler does not have an immediate impact on antecedent retrieval. We must qualify this statement because the effect of gender match at the pronoun was somewhat equivocal in light of the pairwise differences between the Crossover conditions early in the sentence. Even though differences in reading times between the two Crossover conditions were comparable at the pre-pronoun complementizer directly before the pronoun, we cannot rule out the possibility that the differences in previous regions carried or influenced the effect of gender match at the pronoun and afterward. We ran Experiment 2c in order to determine whether we would replicate the gender-mismatch effect, while eliminating baseline differences.

### 3.4 Experiment 2c

#### 3.4.1 Participants

Sixty-two participants (mean age = 34.03, 31 male) were recruited through the Amazon Mechanical Turk marketplace and paid $4.50 for their participation. Participants' IP addresses were restricted to those within the continental US.

#### 3.4.2 Procedure and materials

We used a procedure that was broadly similar to the procedure in Experiment 2b with minor modifications. Sentences were presented using IBEX Farm. Because we were concerned that unmonitored internet participants might be more likely to attend less to short regions, we used a phrase-by-phrase moving window display method in Experiment 2c. We reasoned that lengthening the regions would increase the possibility that participants would dwell on the region containing the pronoun.

#### 3.4.3 Analysis

The analysis procedure was identical to Experiments 1b and 2b. Two participants were excluded from the analysis because their overall comprehension accuracy fell below 70%.

We used the same analysis regions in Experiment 2c as in Experiment 2b except for the pronoun region. Since the pronoun and the post-pronoun were presented as a single unit, they were analyzed together (the *pronoun region*).

#### 3.4.4 Results and discussion

##### Comprehension question accuracy

Mean comprehension question accuracy was 83.8% and did not differ by condition (|*z*| < 1 for all effects).

##### Reading times

**Figure 3:**
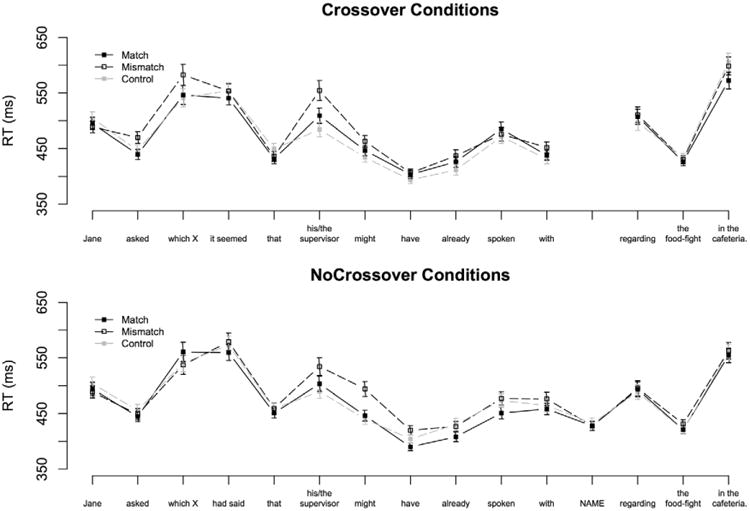
Average phrase-by-phrase self-paced reading times in Experiment 2c. Error bars indicate standard error of the cell means.

Average phrasal RTs by region and condition are plotted in Figure 3. A statistical summary of results in the test conditions is given in Table 9.

There were no significant differences in reading times across conditions at the filler region. The pairwise differences that were observed between the Crossover conditions at the embedded predicate in Experiment 2b were absent, ensuring a stable baseline for two regions prior to the critical pronoun. Reading times at the complementizer were greater in the NoCrossover conditions than in the Crossover conditions (*t* = 3.10, *p* < .01).

At the pronoun region the Pronoun conditions were read more slowly on average than the Control conditions (*t* = –2.48, *p* < .05). Participants read the phrase containing the genitive pronoun and noun faster when the pronoun matched the filler in gender (main effect of GenderMatch: *t* = 2.50, *p* < .05). No GenderMatch × Crossover interaction was observed (*t* < 1), which indicates that the size of the gender-mismatch effect did not differ significantly between the Crossover and NoCrossover conditions.

In the first auxiliary region the Match conditions were read more quickly on average than the Mismatch conditions in the subsequent auxiliary region (*t* = 3.10, *p* < .01). The model revealed a significant GenderMatch × Crossover interaction (*t* = –2.39, *p* < .05), which reflects the fact that there was a significant gender-mismatch effect in the NoCrossover conditions (*t* = –3.88, *p* < .001), but not in the Crossover conditions (*t* < 1). The same pattern of effects, characterized by a GenderMatch × Crossover interaction, persisted into the second auxiliary region (*t* = –2.48, *p* < .05). Reading times in both Crossover conditions were comparable (*t* < 1), but a clear gender-mismatch effect was present in the NoCrossover conditions (*t* = 3.48, *p* < .01). This trend towards facilitation in the Match-NoCrossover condition alone persisted throughout the next three regions.

#### 3.4.5 Discussion

Overall, the results of Experiment 2c are broadly consistent with the results of Experiment 2b. We observed a gender-mismatch effect at the region containing the pronoun in both the Crossover and the NoCrossover conditions. The GenderMatch × Crossover interaction that would characterize Weak Crossover sensitivity was not observed immediately in the same region. Immediately following the pronoun region, we did observe a GenderMatch × Crossover interaction. We take these results to indicate that participants successfully apply the constraint on A-binding at a delay in Weak Crossover configurations. This delayed sensitivity contrasts with the behavioral pattern observed in Strong Crossover sentences, where grammatical sensitivity was apparent immediately at the pronoun. The results suggest that early sensitivity to the unacceptability of the matching wh-filler in Strong Crossover configurations should be attributed to Principle C rather than to the A-binding constraint.

## 4 General discussion

Our primary empirical goal in this study was to assess whether online antecedent retrieval exhibits sensitivity to Crossover configurations. Our theoretical goal was to investigate which aspects of the syntactic representation the parser can use to constrain antecedent retrieval. We achieved this second goal by comparing immediate sensitivity to two different constraints: Principle C (Chomsky 1982) and the constraint on A-binding (Reinhart 1983).

In two offline and three self-paced reading experiments we used a gender-mismatch paradigm to determine if retrieval would access a preceding wh-filler that matched a critical pronoun in gender as a potential antecedent. In each experiment we presented the wh-filler and the pronoun in two different configurations. In one configuration the wh-filler and the pronoun were not in a crossover relation and the matching filler was therefore a grammatically acceptable antecedent for the pronoun. In the second configuration the filler and the pronoun stood in a Crossover relation because the gap associated with the filler followed the pronoun in the linear string. We compared the gender-mismatch effects in the acceptable configurations and crossover configurations. We reasoned that gender-mismatch effects should be smaller or absent in crossover structures than in acceptable structures if the parser was sensitive to the Crossover constraint. On the other hand, if gender-mismatch effects in Crossover configurations were comparable in size to effects in acceptable sentences, we could conclude that the constraints had not applied at the point of antecedent retrieval.

Experiment 1 investigated antecedent retrieval in Strong Crossover configurations, where the critical pronoun both preceded and c-commanded the gap. In Experiment 1a, an offline acceptability judgment study, native speakers rated test sentences as unacceptable when the filler and the pronoun stood in a Strong Crossover relation. In contrast, participants gave high ratings to sentences when the filler and the pronoun were in a grammatically acceptable configuration. These results show that Crossover constraints impact participants' overall judgment of pronoun binding relations, but they do not speak to the question of whether such constraints apply immediately at antecedent retrieval during incremental sentence processing. Experiment 1b used the self-paced reading method to test real-time sensitivity to Strong Crossover configurations at antecedent retrieval. As expected, we found a significant gender-mismatch effect in the NoCrossover conditions immediately following the pronoun: participants read the following region faster when the filler matched the pronoun than when it did not match. This mismatch effect indicates that participants had no trouble retrieving a matching filler when it was an acceptable antecedent. A reliable gender-mismatch effect was not observed in the post-pronoun region in Strong Crossover conditions. The difference in the size of the gender-mismatch effect between the NoCrossover and the Crossover conditions is consistent with the hypothesis that antecedent retrieval displays immediate sensitivity to either Principle C or the A-binding constraint. However, because both constraints apply to Strong Crossover, immediate sensitivity to the configurations does not allow us to determine which constraint was responsible for ruling out the unacceptable filler.

Experiment 2 tested antecedent retrieval in Weak Crossover constructions, where filler-pronoun binding was only ruled out by the argument constraint. Testing Weak Crossover allowed us to measure the contribution of the argument constraint to antecedent retrieval in isolation. We argued that if antecedent retrieval was just as likely to ignore matching fillers in Weak Crossover constructions as in Strong Crossover constructions, we would have evidence that retrieval was sensitive to the argument constraint but not Principle C. If, on the other hand, antecedent retrieval appeared to access matching fillers more readily in Weak Crossover than in the Strong Crossover configurations, then we conclude that sensitivity in Strong Crossover was due to application of Principle C.

The acceptability judgment study Experiment 2a confirmed that participants do not accept matching wh-fillers as antecedents for pronouns that they stand in a Weak Crossover relation with. In two self-paced reading experiments on Weak Crossover we observed a pattern of results that differed substantially from the results of Experiment 1. In Experiment 2b, a word-by-word self-paced reading study, we observed a large gender-mismatch effect immediately at the pronoun in the Crossover conditions. This gender-mismatch effect appeared one region earlier than the gender-mismatch effect in the acceptable NoCrossover conditions. In the post-pronoun region the gender-match effects in the Crossover conditions did not differ significantly from the gender-mismatch effects in the NoCrossover conditions. The results of Experiment 2b provided preliminary evidence that the A-binding constraint might not have an immediate effect on antecedent retrieval. In Experiment 2c, a phrase-by-phrase self-paced reading study, we again observed comparable gender-mismatch effects in both the Crossover and the NoCrossover conditions. Gender-mismatch effects in Weak Crossover were distinguished from mismatch effects in NoCrossover sentences by their duration. Facilitation was long lasting in the NoCrossover conditions: it began at the pronoun and persisted for a number of subsequent regions. The gender-mismatch effect was short-lived in the Weak Crossover conditions: it emerged clearly at the pronoun, but subsided within two regions.

Taken together, the results of Experiments 1 and 2 suggest that Principle C plays an immediate role in constraining the retrieval of potential antecedents for a pronoun. The gender-mismatch effects in Experiment 2 suggest that the argument constraint does not immediately block access to matching fillers in crossover configurations. We therefore conclude that antecedent retrieval must have access, in some form, to the relations between the pronoun and the unseen gap position. We discuss some implications of our findings below.

### 4.1 The scope of syntactic prediction

According to our reasoning, the ability to immediately rule out filler-pronoun binding in Strong Crossover configurations requires the parser to make a decision based on the yet-to-be-seen gap position. We take this result to entail that the parser carries forward commitments about the position of a gap phrase, at least in abstract terms, in advance of receiving bottom-up confirmation of the true gap position. That is, the parser engages in some degree of syntactic prediction during filler-gap processing that extends beyond the immediate phrase.

Our results are broadly compatible with a number of different models of filler-gap processing. They could be modeled by a serial parser that actively projects a gap position for the filler in advance of a verbal head (Fodor 1978; Crain & Fodor 1985; Stowe 1986; Frazier & Flores-D'Arcais 1989; Traxler & Pickering 1996; Nakano et al. 2002; Lee 2004; Aoshima et al. 2009; Omaki et al. 2015). The results could also be accommodated by a parallel parser that draws inferences about the c-command relation between the pronoun and the gap across the distribution of possible continuations after the pronoun was encountered. Knowledge of phrase structure and grammatical rules guarantees, however, that the gap would still fall within the c-command domain of the pronoun in all possible continuations. Moreover a parser that explicitly tracked grammatically necessary c-command relations between objects in the syntactic representation (such as the monotonic parsing model of Sturt & Crocker 1996) would be able to implement Principle C without explicitly committing to a single structural analysis. Models that abstain from making predictions about the position of the gap site in advance of a potentially licensing head (Abney 1989; Goodluck et al. 1991; Pickering & Barry 1991; Pritchett 1992; Pickering & Traxler 2001) would have more difficulty capturing our results.

### 4.2 Implications for cue-based retrieval models

Our results are potentially challenging for cue-based models of antecedent retrieval (e.g. Badecker & Straub 2002; Foraker & McElree 2003; Kush, Lidz & Phillips 2015). Under cue-based models, items are stored in a content-addressable memory and are tagged with features that encode their intrinsic properties (e.g., lexical and morphological information), as well as features that encode aspects of the local syntactic and semantic context at the time that the items are first encountered (McElree, Foraker & Dyer 2003; Lewis, Vasishth & van Dyke 2006; van Dyke & McElree 2011). An item is re-activated at retrieval in proportion to the degree to which its features match a set of retrieval cues provided by a retrieval trigger. For the purposes of antecedent retrieval, an NP is retrieved as a potential antecedent if it matches (at least a subset of) the features of the triggering pronoun.

In the cue-based framework, an item can only be retrieved using cues that were already assigned to that item before the retrieval trigger. It is impossible to encode the pronoun-gap c-command relation that diagnoses a crossover violation as a feature on the filler, because there is no evidence that a pronoun exists downstream when the filler is first encountered. As we discussed in the Introduction, the only property of the preceding context that distinguishes Crossover fillers from acceptable fillers is their non-argument status. A simple procedure that excluded any item that does not bear an [Argument] feature, however, would be unable to make the relevant distinction between Strong and Weak Crossover.

We should note that it is not impossible to devise a feature-based scheme capable of distinguishing fillers in Strong Crossover configurations from those in Weak Crossover configurations. The distinction could be made by a parser that made selective use of the [Argument] feature dependent on construction. In Strong Crossover configurations the feature should be used prominently, so as to exclude fillers, but the feature should play less of a role in Weak Crossover configurations. Such conditional cue use is consistent with a proposal in Kush & Phillips (2014), where it was argued that properties of the retrieval context could allow the parser to minimize the probability of interference by favoring certain cues either through preferential cue-weighting (see Clark & Gronlund 1996; Lewis & Vasishth 2005; van Dyke & McElree 2011), or outright cue exclusion. However, conditional cue use is generally inconsistent with the standard assumption that the parser makes unconstrained use of all potentially relevant cues at once (Badecker & Straub 2002; Jäger et al. 2015). Moreover, such conditional cue use raises an interesting learnability question: on what basis could a parser ever learn the conditioning rule?

### 4.3 Time-course and strength of individual constraints

We have argued that our results provide evidence that knowledge of Principle C has an immediate effect on antecedent retrieval. However, there are different ways in which a constraint could exert an influence on the immediate processing of a pronoun. One important question to consider is whether Principle C excludes consideration of unacceptable matching NPs altogether, or whether it simply reduces the probability that such NPs are retrieved and considered. Many prominent models of antecedent retrieval posit that the various features that characterize acceptable antecedents (e.g., morphological, syntactic, or discourse cues) make independent contributions to the probability of retrieving an NP as a candidate (Arnold et al. 2000; Badecker & Straub 2002; Runner, Sussman & Tanenhaus 2006). Under these models it is possible that a syntactic constraint could lower the probability of retrieving a grammatically unacceptable NP, but it might not block access to an unacceptable NP altogether, if that NP matches other cues (e.g. gender or number). On this view we would expect some degree of partial-match interference from grammatically unacceptable NPs if they match other features with the pronoun.

We failed to observe evidence of facilitatory or inhibitory interference from the matching filler at or directly after the pronoun in the Strong Crossover configurations. Processing of the pronoun was not reliably easier or harder in the Match-Crossover condition compared to the Mismatch-Crossover conditions. These findings contribute to the growing number of studies that have failed to find evidence of facilitatory interference during anaphor resolution: (Kennison, 2003; Sturt 2003; Lee & Williams 2008; Dillon et al. 2013; Chow, Lewis & Phillips 2014; Kush & Phillips 2014; Cunnings, Patterson & Felser 2015; Kush, Lidz & Phillips 2015; though see Parker, Lago & Phillips 2015; Parker & Phillips 2017).

Our findings are consistent with the hypothesis that Principle C effectively blocks retrieval of grammatically unacceptable NPs altogether. We do acknowledge, however, that our results are also consistent with a model in which knowledge of Principle C was implemented as a highly-weighted, but not absolute, constraint on retrieval. We did observe a non-significant trend towards a mismatch effect in the post-pronoun region in Experiment 1, which might constitute suggestive evidence that unacceptable fillers were occasionally retrieved or considered.

It is clear that the argument constraint does not have the same degree of influence over initial processing as does Principle C. If the results from Experiment 2b are taken at face value, it would be reasonable to conclude that the argument constraint has no measurable impact on initial antecedent retrieval. The A-binding constraint appears best characterized as a delayed filter on pronoun resolution processes. The results of Experiment 2c indicate that the delay in applying the constraint is relatively short: consideration of an unacceptable matching wh-filler terminates within two words of the pronoun. However, if we allow for initial constraints to be graded or probabilistic, we cannot be certain that the constraint has no immediate impact. The results are also consistent a model that allows the A-binding constraint to reduce the probability or ease of retrieving the unacceptable filler without restricting access to it altogether. We wish to underscore, however, that even if the A-binding constraint were found to exert some impact on immediate processing, its impact is indisputably not large enough to account for the lack of a gender-mismatch effect in Experiment 1. Therefore, our claim that Principle C has an immediate impact goes unchallenged.

### 4.4 Future directions in processing Crossover

We wish to offer some final remarks on the processing and typology of filler-gap constructions, as well as to sketch some directions for future research. Our studies investigated Crossover sensitivity during the incremental processing of embedded wh*-*questions. Other filler-gap (A′) dependencies, such as relative clause formation, are also subject to Crossover constraints. For example, native speakers judge co-interpretation of *the girl* and *she* (9) to be unacceptable.

(9) *Clint annoyed **the girl_i_** that it had seemed that **she_i_** liked.

Our results establish immediate sensitivity to Strong Crossover when processing embedded wh*-*questions, but they do not establish whether antecedent retrieval would exhibit comparable sensitivity while processing relative clauses (or topicalizations). It is possible that antecedent retrieval would use the geometric relation between the pronoun and the gap to rule out consideration of the filler (*that*, or a covert operator). However, it is also conceivable that the referential status of the relative clause head might interfere with the parser's ability to forego consideration of the illicit dependency. This could happen if antecedent retrieval erroneously contacted *the girl* in an attempt to establish co-reference with a previously-mentioned, matching NP. Future research investigating Crossover configurations in other dependencies has the potential, therefore, to provide a more specific characterization of the mechanisms of antecedent retrieval.

Finally, it has long been acknowledged that the referentiality and specificity of the filler has an effect on the degree to which the grammar tolerates apparent violations of Weak Crossover (see Lasnik & Stowell 1991; Falco 2007). Lasnik & Stowell (1991) discussed how *quantificational* fillers like *who* in (10a) induce Weak Crossover effects, but *non-quantificational* (i.e. referential) fillers like the fronted *the professor* in (10b) do not.

(10)

*Who_i_ do his_i_ students admire ___?
- That professor, his_i_ students admire ___.

Falco (2007) argues that the specificity of a filler also modulates the degree to which it is subject to Weak Crossover effects. Weak Crossover effects are stronger for non-specific fillers (e.g., *who, who the hell*) than for specific fillers (e.g., complex wh-phrases like *which professor*). In light of these accounts, we can ask whether the delay in Weak Crossover sensitivity that we saw in Experiment 2 was due to our using specific wh*-*fillers. Would we see immediate Weak Crossover sensitivity if we used bare wh-words instead? We leave this question to future research.

## 5 Conclusion

We showed that the parser makes immediate use of Principle C when attempting to identify an antecedent for a pronoun in Strong Crossover constructions. Antecedent retrieval did not appear to consider gender-matching wh-fillers that stood in a Strong Crossover configuration to a pronoun. However, we did find evidence that matching fillers in Weak Crossover configurations interferes with antecedent retrieval. These results support a view of antecedent retrieval that integrates inferences made over predicted syntactic structure into constraints on backward-looking processes like memory retrieval. They may also provide novel insight into the scope of long-distance syntactic prediction in English filler-gap processing.

## Figures and Tables

**Table 1:** Example experimental item set from Experiment 1.

NoCrossover-Match	Jane asked which maintenance man ____ had said that **he** already spoke with Donna regarding the food-fight in the cafeteria.
NoCrossover-Mismatch	Jane asked which lunch lady ____ had said that **he** already spoke with Donna regarding the food-fight in the cafeteria.
Crossover-Match	Jane asked which maintenance man it appeared that **he** already spoke with ____ regarding the food-fight in the cafeteria.
Crossover-Mismatch	Jane asked which lunch lady it appeared that **he** already spoke with ____ regarding the food-fight in the cafeteria.
NoCrossover-Control	Jane asked which maintenance man ____ had said that **Dana** already spoke with Jim regarding the food-fight in the cafeteria.
Crossover-Control	Jane asked which maintenance man it appeared that **Dana** already spoke with ____ regarding the food-fight in the cafeteria.

**Table 2:** Average raw and z-scored acceptability ratings from Experiment 1a. Standard error of the mean in parentheses.

*raw ratings*	Match	Mismatch	Control
NoCrossover	4.41 (.18)	2.45 (.15)	4.67 (.16)
Crossover	1.93 (.10)	1.89 (.11)	4.62 (.16)
***z-scores***	**Match**	**Mismatch**	**Control**
NoCrossover	0.53 (.08)	−0.45 (.07)	0.70 (.08)
Crossover	−0.74 (.05)	−0.75 (.06)	0.71 (.08)

**Table 3:** Summary of linear mixed effects regression on z-scored ratings from Experiment 1a. P-values estimated using the Satterthwaite approximation.

	*t*-value
Crossover	9.31***
Pro v. Control	18.07***
GenderMatch	7.52***
Crossover × Pro v. Control	−6.82***
Crossover × GenderMatch	7.16***

*** *p* < .001

**Table 4:** Summary of linear mixed effects models from Experiment 1b. P-values estimated using the Satterthwaite approximation.

	Estimate (s.e.)	*t*-value
*Complementizer*		
Intercept	5.767 (0.03)	190.968
Crossover	0.001 (0.01)	0.137
Pro v. Control	0.019 (0.02)	0.956
GenderMatch	0.003 (0.02)	0.226
Crossover × Pro	0.030 (0.04)	0.808
Crossover × GenderMatch	−0.052 (0.03)	−1.618
*Pronoun region*		
Intercept	5.775 (0.0)	180.233
Crossover	−0.010 (0.0)	−0.728
Pro v. Control	0.055 (0.0)	2.718**
GenderMatch	0.011 (0.0)	0.647
Crossover × Pro	0.034 (0.0)	0.894
Crossover × GenderMatch	0.045 (0.0)	1.300
*Adverb*		
Intercept	5.824 (0.04)	154.118
Crossover	−0.010 (0.02)	−0.609
Pro v. Control	0.015 (0.02)	0.700
GenderMatch	0.057 (0.02)	3.158**
Crossover × Pro	0.026 (0.04)	0.628
Crossover × GenderMatch	0.068 (0.04)	1.865+
*Verb*		
Intercept	5.822 (0.03)	177.173
Crossover	0.001 (0.02)	0.122
Pro v. Control	0.004 (0.02)	0.215
GenderMatch	0.031 (0.02)	1.433
Crossover × Pro	0.027 (0.04)	0.687
Crossover × GenderMatch	0.060 (0.03)	1.766+

+ *p* < .10;

* *p* < .05;

** *p* < .01

**Table 5:** Example experimental item set from Experiment 2. Underscores indicate gap position (not presented to participants). Presentation in Experiment 2b was word-by-word. Vertical bars denote phrase boundaries used in phrase-by-phrase presentation in Experiment 2c.

NoCrossover-Match	Jane | asked | which janitor | (____) had said | that | his supervisor | might | have | already | spoken | with | Donna | regarding | the food-fight | in | the cafeteria.
NoCrossover-Mismatch	Jane | asked | which lunch-lady | (____) had said | that | his supervisor | might | have | already | spoken | with | Donna | regarding | the food-fight | in | the cafeteria.
Crossover-Match	Jane | asked | which janitor | it seemed | that | his supervisor | might | have | already | spoken | with | (____) regarding | the food-fight | in | the cafeteria.
Crossover-Mismatch	Jane | asked | which lunch-lady | it seemed | that | his supervisor | might | have | already | spoken | with | (____) regarding | the food-fight | in | the cafeteria.
NoCrossover-Control	Jane | asked | which janitor | (____) had said | that | the supervisor | might | have | already | spoken | with | Donna | regarding | the food-fight | in | the cafeteria.
Crossover-Control	Jane | asked | which janitor | it seemed | that | the supervisor | might | have | already | spoken | with | (____) regarding | the food-fight | in | the cafeteria.

**Table 6:** Average raw and z-scored acceptability ratings from Experiment 2a. Standard error of the mean in parentheses.

*raw ratings*	Match	Mismatch	Control
NoCrossover	4.45 (.17)	3.19 (.16)	4.36 (.16)
Crossover	3.09 (.16)	2.83 (.17)	3.96 (.16)
***z-scores***	**Match**	**Mismatch**	**Control**
NoCrossover	0.49 (.10)	−0.24 (.09)	0.44 (.09)
Crossover	−0.35 (.08)	−0.50 (.08)	0.16 (.08)

**Table 7:** Summary of linear mixed effects regression on z-scored ratings from Experiment 2a. P-values estimated using the Satterthwaite approximation.

	*t*-value
Crossover	6.35***
Pro v. Control	6.57***
GenderMatch	5.19***
Crossover × Pro v. Control	−1.79***
Crossover × GenderMatch	3.74***

*** *p* < .001

**Table 8:** Summary of linear mixed effects models from Experiment 2b. P-values estimated using the Satterthwaite approximation.

	Estimate (s.e.)	*t*-value
*Complementizer*		
Intercept	5.844 (0.03)	173.00
Crossover	−0.015 (0.02)	−0.602
Pro v. Control	−0.058 (0.03)	−2.246*
GenderMatch	−0.003 (0.02)	−0.153
Crossover × Pro	0.015 (0.05)	0.294
Crossover × GenderMatch	0.061 (0.05)	1.347
*Pronoun region*		
Intercept	0.061 (0.03)	175.536
Crossover	0.003 (0.02)	0.142
Pro v. Control	−0.096 (0.03)	−3.449**
GenderMatch	0.040 (0.02)	1.663+
Crossover × Pro	0.070 (0.06)	1.263
Crossover × GenderMatch	0.088 (0.05)	1.814+
*Post-pronoun Noun*		
Intercept	5.892 (0.04)	152.242
Crossover	−0.007 (0.02)	−0.288
Pro v. Control	−0.079 (0.03)	−2.396*
GenderMatch	0.079 (0.03)	2.746**
Crossover × Pro	−0.055 (0.07)	0.825
Crossover × GenderMatch	0.028 (0.06)	0.477
*First auxiliary*		
Intercept	5.890 (0.04)	165.442
Crossover	−0.007 (0.02)	−0.325
Pro v. Control	−0.073 (0.03)	−2.332*
GenderMatch	0.003 (0.03)	0.094
Crossover × Pro	0.061 (0.06)	1.040
Crossover × GenderMatch	0.097 (0.05)	1.869+

+ *p* < .10;

* *p* < .05;

** *p* < .01

**Table 9:** Summary of linear mixed effects models from Experiment 2c. P-values estimated using the Satterthwaite approximation.

	Estimate (s.e.)	*t*-value
*Complementizer*		
Intercept	6.002 (0.04)	140.087
Crossover	0.051 (0.02)	3.098**
Pro v. Control	0.032 (0.02)	1.274
GenderMatch	0.012 (0.02)	0.605
Crossover × Pro	−0.082 (0.04)	−1.872+
Crossover × GenderMatch	0.050 (0.04)	1.319
*Pronoun region*		
Intercept	0.061 (0.06)	107.691
Crossover	0.003 (0.02)	0.195
Pro v. Control	−0.057 (0.02)	−2.479*
GenderMatch	0.050 (0.02)	2.470*
Crossover × Pro	−0.007 (0.05)	−0.162
Crossover × GenderMatch	0.021 (0.04)	0.513
*First auxiliary*		
Intercept	6.005 (0.05)	124.179
Crossover	0.023 (0.01)	1.607
Pro v. Control	−0.047 (0.02)	−2.158*
GenderMatch	0.055 (0.02)	3.104**
Crossover × Pro	−0.051 (0.04)	−1.280
Crossover × GenderMatch	0.083 (0.03)	2.386*
*Second auxiliary*		
Intercept	5.910 (0.04)	137.505
Crossover	0.013 (0.01)	0.915
Pro v. Control	−0.015 (0.02)	−0.845
GenderMatch	0.038 (0.02)	2.449*
Crossover × Pro	−0.024 (0.04)	−0.679
Crossover × GenderMatch	0.077 (0.03)	2.480*

+ *p* < .10;

* *p* < .05;

** *p* < .01

## Footnotes

1 As their name suggests, Weak Crossover configurations are often described as somehow less marked in introspective judgments than Strong Crossover violations (see Wasow 1972; Cole 1974 for early discussion).

2 This update procedure appears inconsistent with the assumptions of syntactic analyses that do not allow a filler to bind a pronoun directly (Koopman & Sportiche 1982; Reinhart 1983; Sportiche 1985; Ruys 2000). Under these analyses, a filler only binds a trace at the gap site. It is the trace itself that occupies the argument position and binds the later pronoun. Although we have described the procedure in terms that suggest a “direct-composition” approach to filler interpretation and binding (Pickering & Barry 1991; Pollard & Sag 1994; Steedman 2000), it is equally possible to describe the update procedure in terms of traces: Instead of updating the representation of the filler, the parser would simply need to create a representation of a trace, which should then act as a potential binder visible to antecedent retrieval.

3 The first participant was rejected due to low variance in average word-reading times (standard deviation of the participant's mean by-region RT < 30 ms). The standard deviation of the second participant's reading times was abnormally long (>500 ms). Both of these profiles suggest that the participant was not performing the task as instructed.

4 RTs for multi-word regions represent the average over all individual words within those regions.
